# Supplementary material for: Exploring Lysophosphatidylcholine as a Biomarker in Ischemic Stroke: The Plasma–Brain Disjunction
Source: Int J Mol Sci. 2024 Oct 3;25(19):10649. doi: 10.3390/ijms251910649 (PMC11477326; doi:10.3390/ijms251910649)
Supplement: Supplementary file 1 [file ijms-25-10649-s001.zip › ijms-3216321-supplementary.pdf]

Exploring lysophosphatidylcholine as a biomarker in ischemic stroke: the plasma-brain disjunction

Justin Turpin,[1,2,3] Willians Tambo,[2,3,4] Steven Wadolowski,[2,3] Daniel Kim,[2,3,5] Yousef Al-Abed,[3,4] Daniel Sciubba,[1,7] Lance Becker,[3,4,6] Junhwan Kim,[3,6] David LeDoux,[1] Keren Powell,[2,3] Chunyan Li,[1,2,3,4,7]

1 Department of Neurosurgery, Zucker School of Medicine at Hofstra/Northwell, Hempstead, NY, USA
2 Translational Brain Research Laboratory, Feinstein Institutes for Medical Research, Manhasset, NY, 11030, USA
3 Institute of Bioelectronic Medicine, Feinstein Institutes for Medical Research, Manhasset, NY, USA
4 Elmezzi Graduate School of Molecular Medicine at Northwell Health, Manhasset, NY, 11030, USA
5 Boston College, Chestnut Hill, MA, 02467, USA
6 Laboratory for Critical Care Physiology, Feinstein Institutes for Medical Research, Manhasset, NY, USA
7 Donald and Barbara Zucker School of Medicine at Hofstra/Northwell, Hempstead, NY, USA
* Correspondence: cli11@northwell.edu

This file contains supplementary figures 1-3.

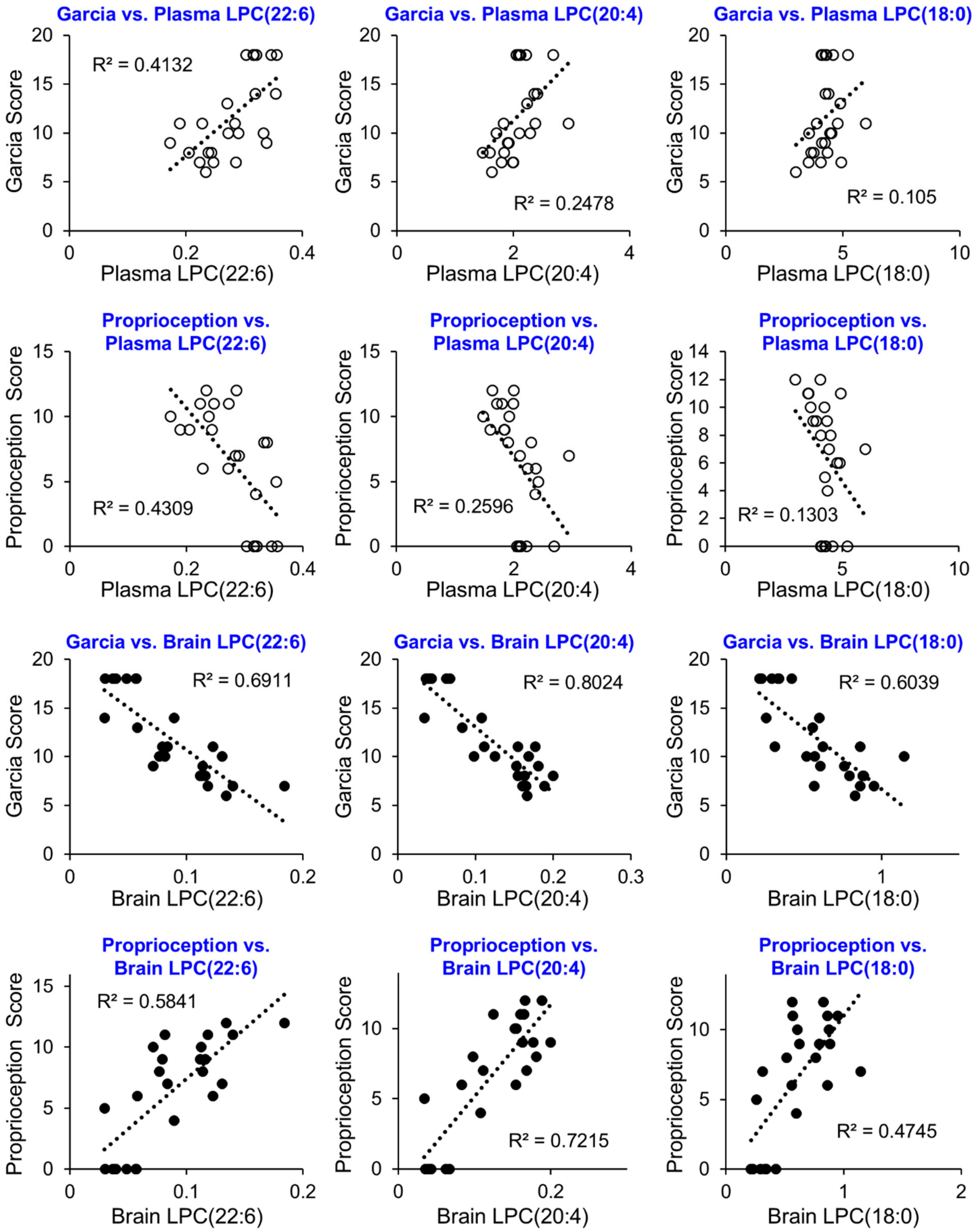

Supplementary Figure S1. Plasma and brain LPC levels correlate with functional outcome. Plasma levels of LPC(22:6), LPC(20:4), and LPC(18:0) exhibit weak to fair correlations with sensorimotor function. Brain tissue levels of LPC(22:6), LPC(20:4), and LPC(18:0) exhibit fairly strong correlations with sensorimotor function.

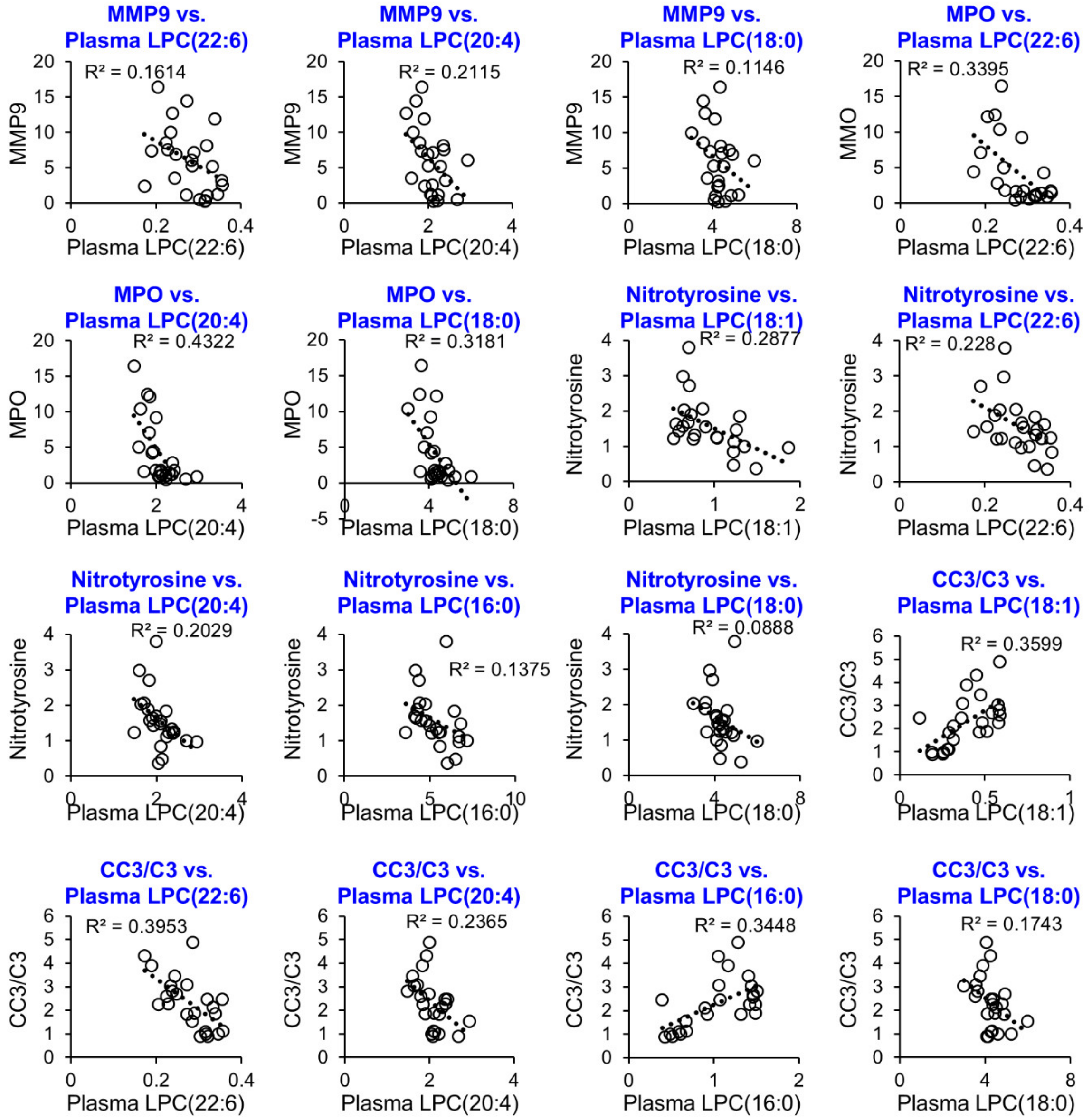

Supplementary Figure S2. Plasma LPC levels correlate with damage markers. Plasma levels of LPC species exhibit weak to fair correlations with MMP9, MPO, Nitrotyrosine, and CC3/C3.

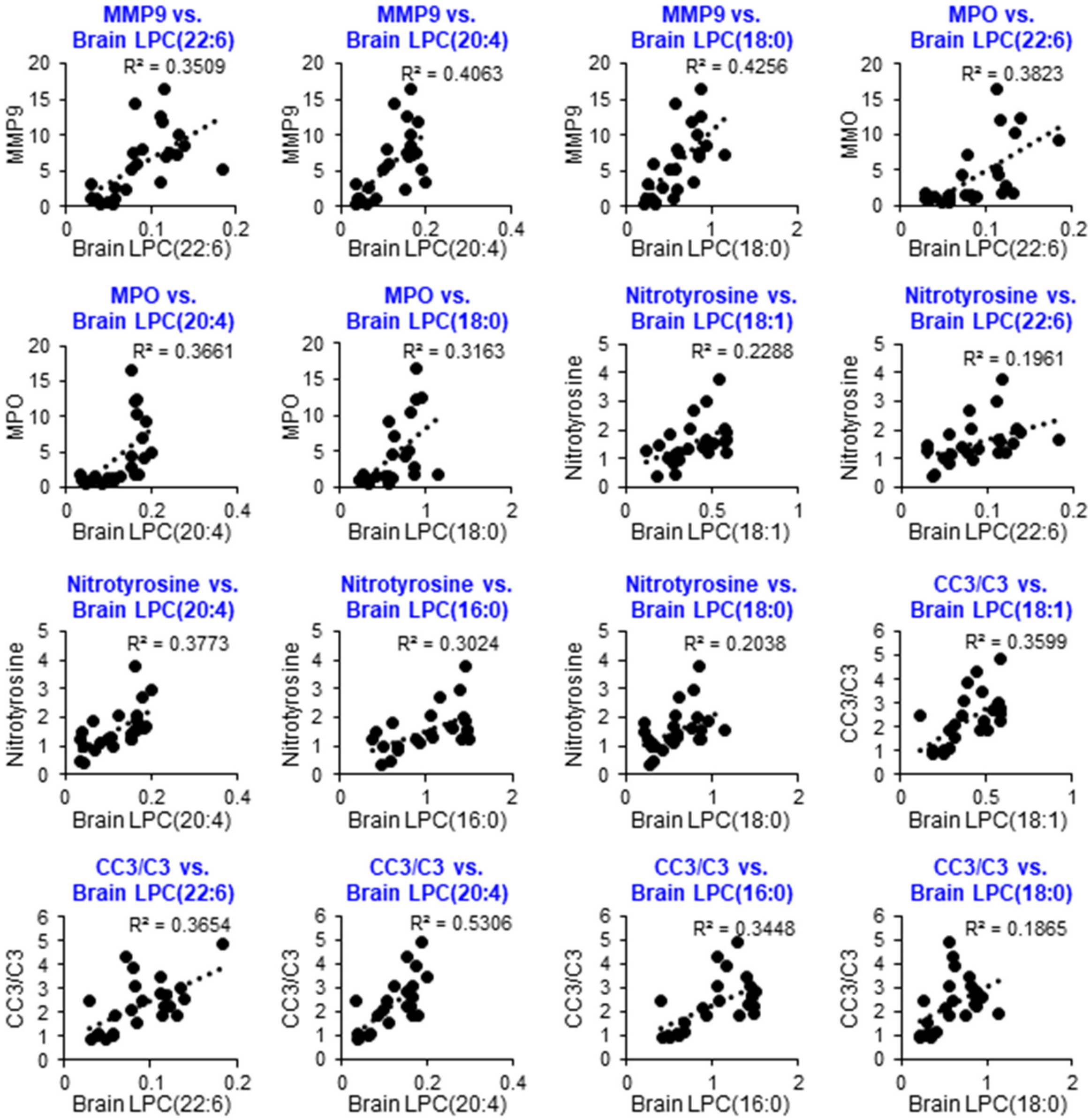

Supplementary Figure S3. Brain LPC levels correlate with damage markers. Brain tissue levels of LPC species exhibit weak to fair correlations with MMP9, MPO, Nitrotyrosine, and CC3/C3.
